# Performance of two serodiagnostic tests for loiasis in a Non-Endemic area

**DOI:** 10.1371/journal.pntd.0008187

**Published:** 2020-05-26

**Authors:** Federico Gobbi, Dora Buonfrate, Michel Boussinesq, Cedric B. Chesnais, Sebastien D. Pion, Ronaldo Silva, Lucia Moro, Paola Rodari, Francesca Tamarozzi, Marco Biamonte, Zeno Bisoffi

**Affiliations:** 1 IRCCS Sacro Cuore Don Calabria Hospital, Center for Tropical Diseases, Negrar, Verona, Italy; 2 Institut de Recherche pour le Développement (IRD), UMI 233-INSERM U1175-Montpellier University, Montpellier, France; 3 Department of Infectious Diseases, Foodborne and Neglected Parasitic Diseases Unit, Istituto Superiore di Sanità, Rome, Italy; 4 Drugs & Diagnostics for Tropical Diseases, San Diego, California, United States of America; 5 Infectious Infectious Diseases and Tropical Medicine Section, Diagnostic and Public Health Department, University of Verona, Verona, Italy; George Washington University School of Medicine and Health Sciences, UNITED STATES

## Abstract

Loiasis, caused by the filarial nematode *Loa loa*, is endemic in Central and West Africa where about 10 million people are infected. There is a scarcity of convenient, commercial diagnostics for *L*. *loa*. Microscopy requires trained personnel and has low sensitivity, while the serodiagnosis is currently not standardized. Individual case management is also important in non-endemic countries to treat migrants, expatriates and tourists. We retrospectively compared the performance of a *Loa* Antibody Rapid Test (RDT) and a commercial ELISA pan-filarial test on 170 patients, 65 with loiasis [8 with eyeworm, 29 with positive microfilaremia, 28 with neither microfilaremia nor history of eyeworm but eosinophilia and history of Calabar swelling (probable loiasis)], 95 with other common parasitic infections and no previous exposure to *L*. *loa* (37 with *M*. *perstans*, 1 with *Brugia* sp., 18 with strongyloidiasis, 20 with schistosomiasis, 5 with hookworm, 4 with *Ascaris lumbricoides* infection, 10 with hyper-reactive malarial splenomegaly), and 10 uninfected controls. The sensitivity of the RDT and of the ELISA were 93.8% (61/65) and 90.8% (59/65), respectively. For the RDT, most of the cross-reactions were observed in patients with *M*. *perstans*: 7/37 (18.9%), followed by 1/10 (10%) with hyper-reactive malarial splenomegaly and 1/20 (5%) with schistosomiasis. None of the 27 subjects infected with intestinal nematodes was found positive at this test. The ELISA is meant to be a pan-filarial assay, and reacted extensively with cases of *M*. *perstans* (95%), as expected, and also in 11/18 (61.1%) patients with strongyloidiasis and in 3/5 (60%) with hookworm infection. The RDT and the ELISA are both highly sensitive for the diagnosis of loiasis. The main difference lies in the extent of cross-reactivity with other parasites. Considering that the RDT is specifically meant for *Loa loa* infection, and its high sensitivity, this test could be a useful tool for the diagnosis of occult loiasis.

## Author summary

*Loa loa* is a filarial worm which infects millions of people living in the forested areas of central Africa. The infection is rarely diagnosed outside Africa, but individual case management is also important in non-endemic countries to treat migrants, tourists and expatriates. Aim of this study was to describe the performance of a Rapid Test and an ELISA pan filarial test for the diagnosis of *Loa loa* infection. Both tests demonstrated good performance for the detection of cases of loiasis. In case of infection with other parasites, the RDT gave less false positive results.

## Introduction

Loiasis, the disease caused by the infection with the filarial nematode *Loa loa*, is transmitted through the bite of tabanid flies of the genus *Chrysops*. It is endemic in Central and West Africa where, according to the most recent estimates, about 10 million people are infected [1].

The adult worms reside in the subcutaneous tissues and in the intermuscular layers, while the progeny, microfilariae (mf), circulate in the blood where they are picked up by the vector during a blood meal, allowing the perpetuation of the biological cycle [2].

The two most specific signs of infection are transient oedemas known as “Calabar swellings”, and the movement of the adult worm under the conjunctiva (“eye worm”) [2]. These manifestations are major reasons for people seeking medical advice in endemic areas [3]. Loiasis is still regarded as a benign condition even if a recent a study found an association between high *L*. *loa* microfilarial density (MFD) and increased mortality risk [4]. Importantly, severe adverse events (SAEs) after administration of diethylcarbamazine (DEC) and ivermectin (IVM) may occur in individuals with high *L*. *loa* MFD [5]. Hence, it is mandatory to rule out this infection before treatment with these drugs, or at least to quantify the number of circulating mf.

From a global health perspective, the World Health Organization (WHO)-driven programmes for the elimination of onchocerciasis rely on mass drug administration (MDA) of IVM. In areas where onchocerciasis is meso or hyperendemic (prevalence of nodules >20%) and loiasis is co-endemic, MDA is possible but a specific surveillance system has to be put in place because of the risk of IVM-induced SAEs. When hypoendemic onchocerciasis coexists with loiasis, a test and treat strategy can be used, so that the whole population is screened to identify those individuals who are at risk of SAEs [6]. Individual case management is also important both in endemic and non-endemic countries to treat migrants, tourists and expatriates. For instance, the Department of Infectious/Tropical Diseases (DITM), IRCCS Sacro Cuore Don Calabria Hospital, Negrar, Verona, Italy has diagnosed and treated at least 120 patients with loiasis in the last 30 years [7]. Clinical diagnosis is possible, although difficult: the passage of the adult worm under the conjunctiva is sporadic, and oedemas are also transient and have been reported in infections with another filarial species, *Mansonella perstans* [8,9]. The identification of circulating *L*. *loa* mf by microscopy confirms the diagnosis of infection. Unfortunately, this method has low sensitivity. *L*. *loa* microfilaraemia in the peripheral blood shows a diurnal periodicity, with maximum MFD found between 10 am and 4 pm; therefore the time of sampling influences the sensitivity of microscopy [2]. Further, mf are not present in the blood during the pre-patent period (4–8 months after the infective bite by the vector) and about 40% of the infected individuals present a so-called “occult loiasis”, i.e. they will never show any mf in the peripheral blood, due to a genetic predisposition [10]. There is a scarcity of convenient, commercial diagnostics for *L*. *loa*. Microscopy is time-consuming and requires trained personnel. A cell-phone based microscopy has been described to simplify the measurement of circulating mf and identify individuals at risk of IVM-induced adverse events [11]. Molecular biology techniques such as PCR or LAMP (Loop-mediated Isothermal Amplification) have been developed, but have not entered in clinical routine practice yet [12–15].

The serodiagnosis of loiasis is currently not standardized. In published clinical-based reports, serodiagnosis was carried out using a variety of techniques and both homologous and heterologous antigen sources [7,16–22]. The specificity of the antigenic preparations to detect antibodies against *L*. *loa* has not been widely explored, but a variable level of cross-reactivity is generally observed in individuals with other filarial infections and also in case of infections with other helminthiases such as strongyloidiasis [23]. Sensitivity is also variable, with only a proportion of infected patients, as assessed by presence of circulating mf or PCR, who have detectable antibodies. Using sera from monkeys experimentally infected with *L*. *loa*, Klion and colleagues identified an antigen, *Ll*-SXP-1, which was poorly sensitive (56%) for human loiasis but highly specific (98%) when tested in an ELISA-IgG4 assay using sera from patients infected with other filarial worms and nematodes [24]. Burbelo and colleagues subsequently evaluated this recombinant antigen using an IgG Luciferase Immuno-Precipitation System (LIPS) improving both sensitivity (67%) and specificity (99%) [25]. Finally, Pedram and colleagues developed a recombinant *Ll*-SXP-1 based lateral flow rapid diagnostic test (RDT) with further improved sensitivity (94%), and acceptable specificity (82%-100% depending on the control panel tested) [26]. This test is the first *L*. *loa*-specific available assay. A RDT could be useful both in *L*. *loa* endemic areas (for loiasis mapping or for a first-step screening of individuals who should be excluded from MDA with IVM) and in non-endemic settings, where it could speed up and improve diagnosis, even in absence of highly-skilled parasitologists.

The primary objective of this study was to evaluate and compare the performance of the novel lateral flow RDT for loiasis and of a commercial ELISA test for filarial infections in a non-endemic setting, in a mixed population of migrants, tourists and expatriates.

Secondary objectives were to assess the diagnostic concordance between the two tests and to assess the reproducibility of the RDT.

## Methods

This diagnostic accuracy study was carried out in the laboratory of the DITM. The study protocol received ethical clearance from the Ethics Committee for Clinical Experimentation of Verona and Rovigo on February 13, 2019 (protocol number 8575).

The RDT was applied on archived serum specimens kept frozen at -80°C at the same institution, from the day of the sample collection till the day of test execution. The results of the ELISA test were already available as part of the routine tests performed at the time of diagnosis.

### Participant selection and study groups

The study was carried out using fully anonymized serum samples available at the DITM and collected between 1994 and 2018. Criteria for inclusion were: presence of a signed informed consent for using the biological sample for research purpose; availability of clinical and demographic information relevant for the study (including age, sex, symptoms, country where the infection was likely acquired, eosinophil count). Expatriates are defined as people who stayed in an endemic country for more than six consecutive months. Three different groups of sera were constituted:

- Group I (“Cases”) was composed by patients diagnosed with *L*. *loa* infection according to one of the following case definitions:
  - “eyeworm”: patients with negative microfilaraemia but with documented passage of an adult *L*. *loa* under the conjunctiva;
  - “mf-positive”: patients with circulating *L*. *loa* mf;
  - “probable loiasis”: patients with neither eyeworm nor microfilaraemia, but presenting eosinophil counts ≥500/μL and history of Calabar swelling in the 2 months preceding the blood sampling.
- Group II (Non-endemic controls): samples collected from patients born and resident in non-endemic areas for loiasis, with no travel history to endemic countries and presenting to the hospital with other clinical conditions, excluding any parasitic infection; the sera were present in the biobank of the DITM and retrospectively tested with both the ELISA and RDT for this study.
- Group III included samples collected from subjects with no previous exposure to *L*. *loa* (i.e. who had never lived in countries where loiasis is endemic: Nigeria, Cameroon, Gabon, Equatorial Guinea, Congo, Democratic Republic of Congo, Central African Republic, Angola, Uganda, South Sudan, Chad), but infected with other common parasitic infections, which may be of concern for cross-reactivity:
  - patients with circulating *M*. *perstans* or *Brugia* sp. mf;
  - patients with strongyloidiasis, diagnosed by presence of larvae in stool (by routine microscopy of formol-ether concentrated feces [FECF] or stool culture) and/or high titre (>1:160) positive serology (in-house immunofluorescence antibody test [IFAT]) [27];
  - patients with schistosomiasis, diagnosed by microscopy of FECF or microscopy of filtered urines;
  - patients with hookworm infection diagnosed by microscopy of FECF;
  - patients with *Ascaris lumbricoides* infection diagnosed by microscopy of FECF;
  - patients from sub-Saharan Africa with hyper-reactive malarial splenomegaly (anti-malarial antibody titre >1:160, IFAT-Biomérieux);

### Test methods

*L*. *loa*, *M*. *perstans* and *Brugia* sp. mf were detected with leukoconcentration method processing 13 mL of venous blood, according to the routine procedure followed in our laboratory; in addition, the MFD was assessed by examining Giemsa-stained thick smears, prepared with 100 μL of blood.

The Loa Antibody Rapid Test (Drugs & Diagnostics for Tropical Diseases, San Diego, CA, USA) detects human IgG against a 148-aminoacid sequence of *Ll*-SXP-1, a protein with 51–53% sequence identity with *Wuchereria bancrofti* and *Onchocerca volvulus*, the two most clinically relevant filarial species [26]. When read with the naked eye, the RDT is geared towards high sensitivity (94%) and medium specificity versus other filariae (82–88%) [26]. An optional companion reader, or an inexpensive visual score card, can be used to set a threshold of test line intensity above which the assay is considered to be positive, as would be the case in an ELISA. Depending on the selected threshold, the balance between sensitivity and specificity can be adjusted towards a less sensitive but more specific assay, e.g. 71% sensitivity and 96–100% specificity vs. other filariae. The intensity of the test line does not correlate with the MFD, only with the probability that the infection is due to *L*. *loa* rather than another filaria. In this study, the assay was read with the naked eye, in favour of high sensitivity. The RDT was run within 24 hours from thawing of sera. Two lab technicians independently read the results of the RDT at 20 minutes from execution, and reported a qualitative result (positive/negative/indeterminate) on an electronic sheet. Indeterminate results were reported as such in order to evaluate of the ease of interpretation of the RDT. Laboratory staff involved in the study were blinded to the results of the ELISA (comparator) and of any other lab test previously performed.

The commercial ELISA kit using *A*. *viteae* antigens (Bordier Affinity Products, Crissier, Switzerland) was used as per manufacturer instructions [28]. This test is not specific for single filarial species, and detects IgG against various filarial nematodes affecting humans. A Serological Index (SI) is calculated as per manufacturer’s protocol and a SI ≥ 1 is considered positive. Literature reported test’s accuracy is (a) a sensitivity of 95% in patients with filariasis (certain or probable); (b) a specificity of 98% in blood donors, and of 69% in patients with other parasitic infections [28]. This test had been previously evaluated at the DITM, in association with other parameters (eosinophilia and microfilaraemia), for the follow-up of the patients with *M*. *perstans* and *L*. *loa*, highlighting a seroconversion within 20 months from first treatment [29]. It was chosen as a comparator as part of the present study because it is routinely used at the DITM and in most French hospitals for the screening of patients with suspected filarial infection. The diagnostic algorithm at the DITM entails examination for the presence of microfilaraemia in patients positive to the ELISA, which enables the identification of the filarial species [29].

The results of both tests (RDT and ELISA) were entered in an electronic database set up for the study and protected by a password. The principal investigator monitored the data entry and was in charge of validation in case of discrepancies.

At the time of writing, the cost of the Bordier ELISA is US $ 10–11 per patient, depending on the sales volumes. The Loa Antibody Rapid Test costs $ 3–4 per patient, depending on the sales volumes. The test can be used without additional equipment, as we have done in this article. In addition, the manufacturer proposes an optional companion reader ($1,000) to adjust the balance between sensitivity vs specificity. As a cost-effective alternative to the reader, the manufacturer also proposes a scorecard for simple visual scoring. The scorecard is not only cheaper but also easier to ship internationally and does not require maintenance. Herein, we have not experimented with either the reader or the scorecard.

### Data analysis

The sample size of this study was determined by the available number of archived specimens. The results were summarized using descriptive statistics. Diagnostic estimated parameters were reported with exact 95% confidence intervals (CI) and statistical significance level was fixed at 5%. Both statistical methods and plots were used to assess concordance between tests or readers (Cohen’s kappa coefficient).

The diagnostic performance of the RDT, according to the two readers, was reported as frequencies, from which sensitivity and specificity were derived comparing them to the known diagnosis. As an exploratory analysis, test accuracies were further assessed stratifying the population by infection type. Data analysis was performed using SAS^®^ software version 9.4.

## Results

### Description of the study groups

The sera were collected from 170 patients. Sixty-five belonged to Group I (confirmed or probable loiasis), 10 to Group II (no history of travel/residence in *L*. *loa* endemic countries and admitted for other reasons) and 95 to Group III (no history of travel/residence in *L*. *loa* endemic countries but infected with other helminths/parasites that can be co-endemic with *L*. *loa*). Thirty-eight were females and 132 males. The median age of females was 42 years (inter-quartile range, IQR = 28–57) and that of males was 26 years (IQR = 21–43.5). One hundred nineteen patients (70%) were migrants, 38 (22%) were expatriates and 13 (8%) were tourists. Among the 65 patients of Group I, 8 had a history of eye worm, 29 presented *L*. *loa* mf, and 28 had a “probable loiasis”. Group II included 10 negative controls. The 95 subjects of Group III included 37 with *M*. *perstans* microfilaraemia, one with *Brugia* sp. microfilaraemia, 57 with a different parasitic infection that was tested for possible cross-infection (*A*. *lumbricoides*, hookworm, *Schistosoma* sp., *S*. *stercoralis* or hyper-reactive malarial splenomegaly).

**Fig 1:**
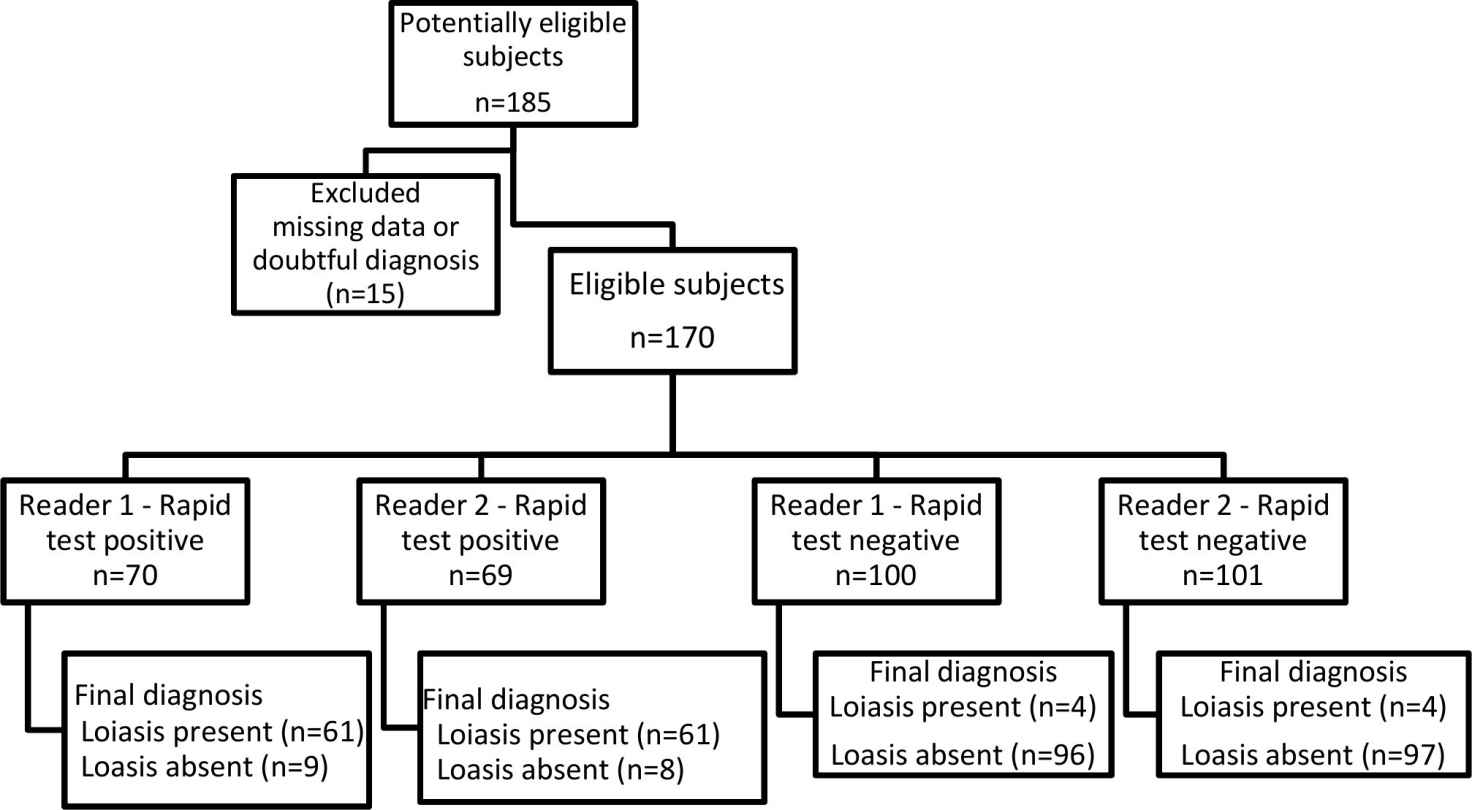
The study flow of the RDT in relation to the final diagnosis.

The study flow (of the RDT) in relation to the final diagnosis is reported in Fig 1 (Flow chart).

The agreement between the two readers was excellent (kappa’s coefficient = 0.99, 95% CI 0.96–1). Only one subject out of 170 had discordant RDT results (indeterminate versus positive). This subject was from Senegal, was co-infected with *M*. *perstans* (103 mf/mL) and *S*. *stercoralis*, and had an ELISA SI of 1.22.

### Sensitivity and specificity of the RDT and of the ELISA test

Table 1 shows the sensitivity of the two tests calculated against Group I, and in each of the sub-groups (patients with microfilaremia, eye worm and probable loiasis). Among the four subjects with a “probable loiasis” who were “missed” by the RDT, two were also negative at the ELISA test, with SI of 0.44 and 0.58. None of these four subjects was a migrant (three were expatriates and one was a tourist). Two were co-infected with *S*. *stercoralis* and one with *Schistosoma* sp., and their eosinophil counts were 1,240, 1,430, 1,830 and 4,770/μL.

**Table 1:** Sensitivity of the RDT and ELISA test by infection type.

	ELISA		RDT	
	No. of positive samples	Sensitivity (95% CI)	No. of positive samples	Sensitivity (95% CI)
Patients with microfilaraemia (n = 29)	27	93.1% (77–99)	29	100% (88.1–100)
Patients with eye worm (n = 8)	6	75.0% (35.0–97.0)	8	100% (63.1–100)
Patients with probable loiasis (n = 28)	26	92.9% (76.5–99.1)	24	85.7% (67.3–96.0)
All patients with confirmed and probable loiasis (n = 65)	59	90.8% (81.0–96.5)	61	93.8% (85.0–98.3)

Table 2 shows the specificity of the two tests calculated against Group II and III. For the RDT, most of the cross-reactions were observed in patients with *M*. *perstans* microfilaraemia: 7/37 (18.9%), followed by 1/10 (10%) with hyper-reactive malarial splenomegaly and 1/20 (5%) with schistosomiasis. None of the 27 subjects infected with intestinal nematodes was found positive at this test. In 11/18 (61.1%) patients with strongyloidiasis and in 3/5 (60%) with hookworm the ELISA test resulted positive. The ELISA (panfilarial) test was also positive in 35/37 samples from patients with *M*. *perstans* infection. No false positive results were observed when testing the 10 sera of patients with no parasitic infections.

**Table 2:** Specificity of the RDT and ELISA test by infection type.

	No. of ELISA positive samples (specificity; 95% CI)	No. of RDT positive samples (specificity; 95% CI) Reader 1	No. of RDT positive samples (specificity; 95% CI) Reader 2
Negative Controls Group II (n = 10)	0 (100%; 69.2–100)		
Patients of Group III (n = 95)	51 (46.3%; 36.0–56.9)	9 (90.5%; 82.8–95.6)	8 (91.6%; 84.1–96.3)
Patients of group III with hyper-reactive malarial splenomegaly (n = 10)	0 (100%; 69.2–100)	1^a^ (90.0%; 55.6–99.8)	
Patients of group III with *Mansonella* (n = 37)	35 (^b^)	7 (81.1%; 64.8–92.0)	6 (83.8%; 68.0–93.8)
Patient of group III with *Brugia* (n = 1)	1 (^b^)	0 (100%; 2.5–100)	
Patients of group III with *Schistosoma* (n = 20)	1 ^c^ (95%; 75.2–99.9)		
Patients of group III with *Strongyloides* (n = 18)	11 (38.9%; 17.3-64-3)	0 (100%; 81.5–100)	
Patients of group III with *Ancylostoma* (n = 5)	3 (40%; 5.3–85.3)	0 (100%; 47.8–100)	
Patients of group III with *Ascaris* (n = 4)	0 (100%; 39.8–100)		

^a^ correspond to the same patient (a migrant from Togo), not tested for mf, with an ELISA SI at 0.20.

^b^ specificity was not reported as ELISA is a pan-filarial test.

^c^ correspond to the same patient (a migrant from Senegal), not tested for mf, with an ELISA SI at 2.05.

According to reader 1, the sensitivity of the RDT was 93.8% (95% CI 85–98.3), the specificity 91.4% (95% CI 85–96). According the reader 2, the sensitivity was of 93.8% (95% CI 88–99.7), the specificity 92.4% (95% CI 85.5–96.7).

## Discussion

This is the first publication describing the performances of two serological assays for *L*. *loa* using the same sample collection to allow for direct comparison between an ELISA test which is not specific for a single filarial species, and the RDT specific for loiasis. The agreement between readers of the RDT was excellent as only one sample had a different result between the two lab technicians. The accuracy of the RDT was very good. In particular, the test was positive for all cases of confirmed loiasis. The sensitivity was lower in case of probable loiasis (85.7%). As patients with eye worm and/or microfilaraemia do not need further tests for confirmation of loiasis, this RDT would be most useful for clinical decision-making of “patients with probable loiasis”. However, the sensitivity for the latter group was not as high as for the other two categories of loiasis. This can be due for instance to the inclusion of false positive cases (in particular two cases with negative RDT and positive ELISA that might have cross-reacted with *Strongyloides stercoralis*). For this reason, sensitivity and specificity of a new diagnostic test for *L. loa* should be ideally assessed using PCR as the reference standard. Actually this is a limitation of our study, but retrospectively it was not possible to carry out PCR due to unavailability of whole blood. Overall, the sensitivity of the RDT was high (93.8%), i.e. very similar to the value (94%) reported by Pedram et al. [26]. The sensitivity of the ELISA was also high (90.8%) and in the same range as the rapid test when taking into account the 95% confidence intervals. The denominators for specificity were different for the two tests, in consideration of the fact that the Group III patients were not subjected to microfilaremia research, so we cannot exclude the presence of *M*. *perstans* mf. Even after excluding the other filarial infections from the denominator, the ELISA presented several cross-reactions (in particular with *S*. *stercoralis*) that affected the specificity (77.6%). A smaller proportion of false positive results was observed for the rapid test, which cross-reacted mostly with *M*. *perstans*. The original report of the performance of the Loa Antibody Rapid Test was rich in *O*. *volvulus* (n = 99), *W*. *bancrofti* (n = 49), and *S*. *stercoralis* (n = 40) samples, and had fewer cases of *M*. *perstans* (n = 16) with reported specificities of 82–88% [26]. Our sample set was different, with no *O*. *volvulus* samples and only one *W*. *bancrofti* sample, but more sera from people with *M*. *perstans* (n = 37) for which the specificity was 81.1%, confirming the specificity range of the original article [26]. In summary, both assays perform as claimed by their respective manufacturers. The RDT and the ELISA are both highly sensitive. The main difference lies in the extent of cross-reactivity with other parasites; the RDT cross reacts when the control population is composed of subjects infected with *M*. *perstans* (18.9%) and does not cross react with *S*. *stercoralis*, while the ELISA reacts extensively with cases of *M*. *perstans* (95%), obviously being a pan-filarial test, and *S*. *stercoralis* (61%).

The RDT detects IgG and not the antigen, hence it results positive also in case of past *Loa loa* infections. This can affect the specificity of the test that, differently to the ELISA, and cannot be recommended for the post-treatment follow-up.

**Fig 2:**
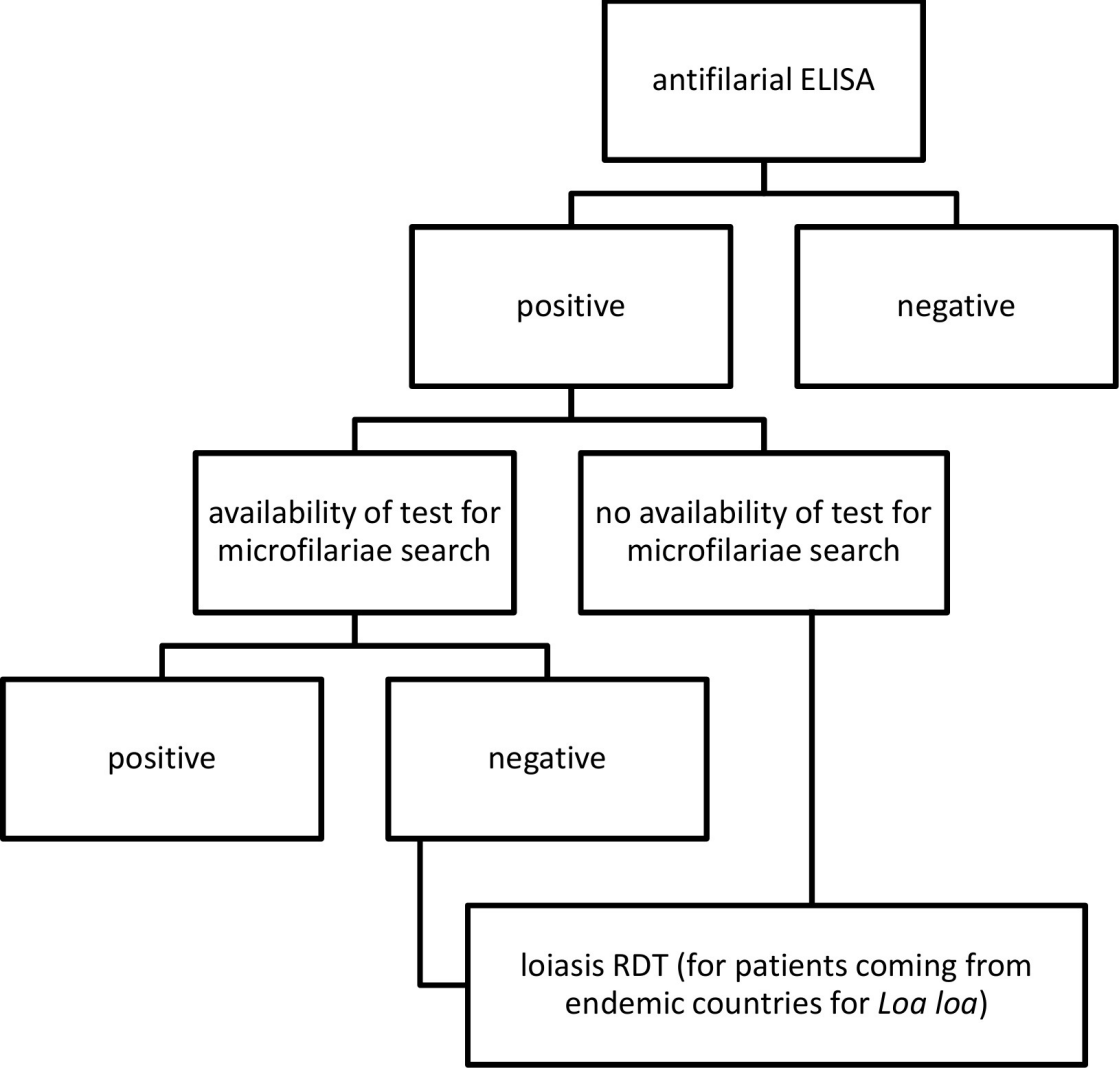
Proposed algorithm for diagnosis of filariasis in non-endemic countries.

The Loa rapid test is a Research Use Only device intended primarily for epidemiological purposes and has not been approved at this point for individual case management. The RDT appears to be suitable for mapping *L*. *loa* prevalence in endemic countries. From a practical standpoint, we found it easy to run, reproducible, and convenient for use at the point of care. However, the RDT has the potential to be useful for a Tropical Diseases Center in a non-endemic country for loiasis, both for centers that assess microfilaraemia and for those that do not (Fig 2).

In case of availability of mf detection, the RDT might be carried out in case of negative microfilaraemia to confirm probable loiasis cases, while in case of unavailability of mf detection, the RDT might be carried out in case of positive ELISA test to differentiate a case of loiasis from other filariasis. Our proposed algorithm does not include costs of the tests, as a cost-effectiveness was not among the purposes of this study.

With the shown high sensitivity, this RDT can replace the microscopy in facilities that are not familiar with loiasis, as all microfilaraemic patients were detected by this RDT. However, as the SAE is related to the burden of mf and the RDT band strength does not correlate to MFD in any case it is necessary to assess microscopy before treatment. This aspect is very important considering that the treatment for *L*. *loa* is different from that of *M*. *perstans* infection. For loiasis the treatment of choice is DEC [30] or IVM + ALB [31] in case of DEC unavailability. Instead, for *M*. *perstans* infection the treatment of choice seems to be doxycycline [32,33], although the treatment usually preferred in European countries is still mebendazole.

## Conclusions

The novel lateral flow RDT has proven to be an accurate and user-friendly tool for the diagnosis of *L*. *loa* infection. While some cross-reactivity with *M*. *perstans* should be taken into account when considering its potential application as a screening tool in endemic areas, on the other hand this new test appears to be promising in the of non-endemic setting, where it could be included in a management algorithm.
